# Phase II Study of ENZAlutamide Combined With Hypofractionated Radiation Therapy (ENZART) for Localized Intermediate Risk Prostate Cancer

**DOI:** 10.3389/fonc.2022.891886

**Published:** 2022-07-14

**Authors:** Pedro C. Lara, Juan I. Rodríguez-Melcón, Amalia Palacios-Eito, Antonio Lozano, Asunción Hervás-Morón, Elena Villafranca, Alfonso Gómez-Iturriaga, Gemma Sancho, Xavier Maldonado

**Affiliations:** ^1^ Canarian Comprehensive Cancer Center, San Roque University Hospital, Fernando Pessoa Canarias University, Las Palmas, Spain; ^2^ Radiation Oncology. Dr. Negrín University Hospital, Las Palmas, Spain; ^3^ Radiation Oncology, Reina Sofía University Hospital, Instituto Maimónides de Investigación Biomédica de Córdoba, Córdoba, Spain; ^4^ Radiation Oncology, Virgen de la Arriexaca University Hospital, Murcia, Spain; ^5^ Radiation Oncology, Ramon y Cajal University Hospital, Madrid, Spain; ^6^ Radiation Oncology, Navarra University Hospital, Pamplona, Spain; ^7^ Radiation Oncology, Cruces University Hospital, Biocruces Bizkaia Health Research Institute, Barakaldo, Spain; ^8^ Radiation Oncology, San Creu I San Pau University Hospital, Barcelona, Spain; ^9^ Radiation Oncology, Vall d´Hebron University Hospital, Barcelona, Spain

**Keywords:** prostate cancer, intermediate risk, enzalutamide monotherapy, hypofractionated, radiotherapy

## Abstract

**Background:**

Intermediate-risk prostate cancer (PCa) is usually treated by a combination of external beam radiation therapy (EBRT) and a short course of androgen deprivation therapy (ADT). ADT is associated with multiple side effects, including weight gain, loss of libido, and hot flashes. In contrast, anti-androgen monotherapy is generally better tolerated in spite of higher rates of gynecomastia.

**Objective:**

This study assessed the effectiveness of enzalutamide monotherapy combined with hypofractionated EBRT (Hypo-EBRT) for treating intermediate risk prostate cancer.

**Method:**

This trial was a multicenter, open-label phase II study of 6 months of enzalutamide monotherapy combined with Hypo-EBRT for intermediate-risk prostate cancer. Hypo-EBRT was initiated 8–12 weeks after initiating enzalutamide. The primary endpoint was PSA decline >80% measured at the 25th week of enzalutamide administration. Secondary end-points included assessment of toxicity, changes in anthropomorphic body measurements, sexual hormones, and metabolic changes.

**Results:**

Sixty-two patients were included in the study from January 2018 to February 2020. A PSA decline of >80% was observed in all evaluable patients at the end of enzalutamide treatment and 92% achieved PSA values under 0.1 ngr/ml. All patients remain in PSA response (<80% reduction of the initial values) 6 months after the end of enzalutamide treatment. The most frequent adverse events were hypertension, asthenia, and gynecomastia. There were no significant changes in bone density, body mass index (BMI), or patient-reported outcomes (PROs).

**Conclusion:**

Enzalutamide monotherapy is very effective along with hEBRT in reducing PSA levels for patients with intermediate-risk prostate cancer. Longer follow-up is needed to confirm the potential use of this combination in future randomized trials.

## Introduction

Radiation therapy (RT) is the standard treatment for localized prostate cancer patients (1). When external beam radiotherapy (EBRT) is used, conventionally fractionated external beam RT (cEBRT) with total escalated doses of 75.6–79.2 Gy (2) is usually prescribed.

Due to the favorable α/β ratio of prostatic cancer, as compared to the surrounding normal tissues (3), the use of hypofractionated schedules would be of interest. For patients, hypofractionated EBRT (Hypo-EBRT) is very convenient, as it reduces the treatment time, improves access to treatment, and lowers the treatment cost (4). Hypo-EBRT administered in 4 to 5 weeks had resulted, in an equivalent disease control rate, compared with escalated cEBRT administered at 8 weeks, with similar acute and late toxicity rates in non-inferiority randomized trials (5–7).

Androgen deprivation therapy is usually combined as adjuvant treatment with EBRT in localized and locally advanced prostate cancers (8). Although it is effective in reducing tumor mass and prostate-specific antigen (PSA) levels (9), limitations to the use of adjuvant ADT in these localized tumors mainly derive from the short- and long-term adverse effects (AEs), which may worsen the quality of life of the patient or be potentially harmful (10–12).

Antiandrogens are considered an alternative to ADT along with EBRT. The use in monotherapy of the first-generation antiandrogen, bicalutamide, along with cEBRT, improves survival in prostate cancer patients in very unfavorable situations without resulting in testosterone-suppression-induced side effects (13–15).

Enzalutamide is a second-generation oral androgen receptor (AR) inhibitor (16) that, unlike classical antiandrogens, blocks different steps in the AR signaling pathway (17, 18). In castration resistant metastatic patients, enzalutamide resulted in better clinical outcomes and reduced toxicity when compared with bicalutamide and ADT (19). Enzalutamide plus ADT is approved for treating adult men with castration-sensitive or resistant metastatic prostate cancer (20–23).

The possibility of using enzalutamide as monotherapy has been extensively studied by Tombal et al. (24–26) as the first treatment in patients with localized and metastatic prostate cancer. They chose the PSA response (<80% PSA decline over pretreatment levels) to assess the activity of enzalutamide, according to previous results from prospective studies with the LHRH antagonist degarelix (27). The use of enzalutamide has a better tolerance profile than LH-RH agonists in terms of body mass, lipid profile, or bone density. The quality of life of the patients did not change with the treatment, and from the sexual perspective, the results were similar to those of bicalutamide. As testosterone levels remain elevated during enzalutamide treatment, sexual toxicity is lower than that observed with ADT therapy, but there was a higher rate of disorders related to the breast (24–26).

Therefore, enzalutamide in monotherapy in men with previously untreated prostate cancer produces an adequate level of suppression of the disease as measured by a long and sustained decrease in PSA with less toxicity than LH-RH agonists (26).

Then, if localized intermediate-risk prostate cancer is to be managed with a combination of radiotherapy and hormonal therapy (28), the possibility of improving the toxicity profile of this treatment, using enzalutamide monotherapy, would be of great benefit to these patients with a good prognosis, who should not suffer bothersome undesirable effects.

Enzalutamide monotherapy radiosensitizes prostate cancer cells to radiation (29) by inducing the suppression of DNA repair mechanisms, mainly through non-homologous end-joining repair suppression mediated by DNAPKc proteins (30). This sensitizing effect was also demonstrated in androgen-sensitive and resistant prostate cancer cell lines, animal models, and xenografts on castration-resistant human prostate cancers (31). Enzalutamide provides a stronger radiosensitation than ADT (32) and, furthermore, this effect is more relevant when higher than 2 Gy doses per fraction (29) are used and enzalutamide is administered concurrently with RT (31). This improved effect on concomitant-adjuvant hormonal therapy with radiotherapy has also been observed for standard ADT in the clinical setting (33).

Therefore, if we consider the use of enzalutamide along with radiotherapy for localized prostate cancer, several questions still need to be answered. First, the immediate acute tumor response estimated by PSA decline of combined enzalutamide with the new standard modern Hypo-EBRT. This Hypo-EBRT schedule would favor radiosensitization induced by enzalutamide and improve tumor response. Second, there is no evidence about the possibility of a durable PSA response after cessation of enzalutamide treatment. This issue is of particular interest as it would encourage the development of future trials comparing standard ADT with enzalutamide monotherapy in this particular setting. Third, the toxicity of such a combination and the quality of life of prostate cancer patients are still unknown.

Based on the clinical and biological findings, we analyze for the first time the use of modern hEBRT along with concurrent enzalutamide monotherapy as treatment for localized intermediate-risk prostate cancer.

## Patients and Methods

This open-label, single-arm, phase 2 study was done across 8 recruiting sites in Spain. Patients were enrolled if they were aged 18 years or older; had histologically confirmed localized **(after diagnostic work-up, namely, pelvic MRI and/or abdomen CT-scan and bone-scan)** intermediate risk prostatic adenocarcinoma (defined as PSA 10–20 ng/ml and/or T2b-C and/or Gleason score 7, if all three factors were present, less than 50% of cores were required to be positive); had an Eastern Cooperative Oncology Group (ECOG) score of 0–1, adequate renal/liver function, and normal blood counts.

Exclusion criteria included previous or current hormonal manipulation, prior treatment for prostate cancer, previous radiation therapy for a pelvic tumor, history of cancer in the last 5 years, history of seizure or treatment with antiepileptic drugs. The full inclusion/exclusion criteria are given in **Supplementary Material Table 1**.

All patients provided written informed consent. This study was conducted in accordance with the Helsinki Declaration and the International Conference on Harmonization: Harmonized Tripartite Guideline: Guideline for Good Clinical Practice. The protocol was approved by local institutional review boards of each center, independent ethics committees, and the Anonymized for Review Government Competent Authority in Spain. The trial was registered at ClinicalTrials.gov, NCT01302041.

### Procedures

After a 4-week screening period, the participants were given a study drug-dosing diary for each of the 6 treatment cycles. Each treatment cycle lasted 28 days (4 weeks), while the participant received the study drug enzalutamide orally. Starting on Day 1, all patients will ingest enzalutamide 160 mg/day at the same time each day, without breaks (except as outlined for toxicity), for 6 (28 days ±3 days) cycles. The dose reduction of enzalutamide to 120 mg/day was allowed with the approval of the principal investigator of the study. Patients were instructed to return all unused capsules at each study visit to assess compliance and received the study drug every 28 days ( ± 3 days) for 6 cycles.

In patients suffering from grade 3 or greater toxic side effects that cannot be reduced by the use of standard medical intervention, treatment should be interrupted until these adverse effects improve. Then, patients could restart on a reduced enzalutamide dose with the written approval of the principal investigator of the study.

Between 8 and 12 weeks after starting enzalutamide, the patients were treated with Hypo-EBRT for a duration of 5.5 weeks. Treatment was administered on an outpatient basis. Hypo-EBRT was administered under Image Guided Radiation Therapy (IGRT) technology. The participant centers were required to routinely use IGRT in these patients, either by ConeBeam CT study and/or fiducial markers placed within the prostate. The External Beam Radiation Dose was normalized such that exactly 98% of the PTV (planned target volume) receives the prescription dose and will be scored as per protocol. The maximum allowable dose within the PTV is 107% of the prescribed dose to a volume that is at least 0.03 cc. The minimum allowable dose within the PTV is >95% of the prescribed dose to a volume that is at least 0.03 cc. The EBRT/IGRT protocol delivered a total dose to the PTV (CTV including the prostate and the proximal seminal vesicles with a 4 mm posterior margin, 8 mm lateral margin, and 5 mm margin in all other directions) of 70 Gy delivered in 28 fractions of 2.5 Gy each. The EQD2 (considering the alpha/beta ratio of 1.5 Gy) was 80 Gy ( 34).

Blood samples to establish PSA and circulating hormone levels were collected at screening, at the 4th and 25th weeks, and 1, 3, and 6 months after the end of enzalutamide. All patients had monthly clinical visits during treatment and safety follow-up visits at 1, 3, and 6 months after their last dose of enzalutamide, recording adverse events graded according to the National Cancer Institute Common Terminology Criteria for Adverse Events, version 4.0.

Blood samples assessing renal, liver, and blood counts were performed at screening and monthly until the end of enzalutamide administration. Fasting serum lipids and fasting glucose levels were assessed on samples collected on day 1, the 12th, and the 25th weeks.

Changes in bone mineral density were assessed by a dual-energy X-ray absorptiometry scan on day 1 and the 25th week. HRQoL was assessed with self- administered EORTC QLQ-C30 and EORTC QLQ-PR25 instruments (35, 36) completed by patients on day 1, at the 12th and 25th week, and at the safety follow-up visit 1 month after the end of enzalutamide.

### Outcomes

The primary outcome was PSA response, defined as a decline from baseline in PSA level of 80% or greater at the 25th week, based on the PSA response observed in registration trials of enzalutamide and other hormonal treatments (24, 27). Enzalutamide-induced PSA decline after 1, 3, and 6 months of cessation of enzalutamide treatment for the primary analysis has also been considered a relevant treatment response marker to assess the activity of enzalutamide combined with hypofractionated radiotherapy. Secondary outcomes were, changes from baseline in hormone level, bone mineral density, fasting serum lipids and quality of life. Safety outcomes included the frequency and severity of adverse events as scored by the CTCAE 4.0.

### Statistical Analysis

The primary activity outcome was the proportion of patients with a PSA response at the 25th since the start of enzalutamide and 1, 3, and 6 months after the cessation of enzalutamide treatment. This was calculated as the number of patients with PSA response (≥80% PSA decline from baseline) at the prespecified time-points, divided by the number of patients who started treatment, and presented as the percentage of patients responding. Patients who discontinued enzalutamide treatment were included in the intention-to-treat analysis. Secondary and exploratory outcomes are summarized descriptively.

The primary endpoint for this trial was to assess the number of patients with a more or equal 80% reduction in baseline PSA at the 25th week. We assume a null hypothesis if 70% of patients do not achieve PSA declines of over 80% and a positive hypothesis if more than 85% of the patients achieve such a decline at the 25th week. We aimed for a “maximum” recruiting scenerio, calculating the target evaluable sample size for an alpha = 0.05 and beta = 0.1 error to be 66 patients, resulting in 70 cases of target recruiting size if a 5% patient loss was considered. A second “standard” calculation of the target evaluable sample size for an alpha = 0,05 and beta = 0.2 error, resulted in 47 evaluable patients to be recruited, reaching 50 patients if a 5% loss was considered.

Safety analyses were performed on all patients who had taken at least one dose of the study drug. All reported toxicities were summarized as acute toxicity regardless of attribution by maximum grade and were sorted by the number of patients experiencing the toxicity during the enzalutamide and Hypo-EBRT treatments and until 1 month post-treatment. Late toxicity was recorded at 6 months after cessation of enzalutamide.

Activity analysis was performed according to the “intention to treat” analysis, including patients who had taken at least one dose of study drug and had both pretreatment and at least one activity evaluation after treatment initiation.

The mean, standard deviation, range, and 95% confidence interval of the mean were calculated to describe the quantitative variables. The Shapiro–Wilk (n ≤50) or Kolmogorov–Smirnov (n >50) test was used to verify the normality of the data of the quantitative variables as a function of the sample size. The qualitative variables have been described by means of the absolute frequency, relative frequency, and the CI (95%) calculated using the Clopper–Pearson method. When the sample size is greater than 30, the Student’s t-test has been used for paired data to compare numerical variables at two different moments of time. In the opposite case, and if the variables do not follow a normal distribution, we have used the Wilcoxon test for paired data. A p-value of less than 0.05 is considered significant. The statistical program used was R Core Team 2021, version 4.1.1 ( 37).

### Role of the Funding Source

This is an independent academic study supported by an unrestricted educational grant from Astellas. The authors performed the protocol design, data analysis, interpretation, and preparation of this report. Data analysis was performed by an independent statistician (JMGM). All authors had access to the study data. All decisions relating to the manuscript writing and content were made jointly by the authors, including the final decision to submit it for publication.

## Results

### Patient’s Characteristics

Sixty-two out of the maximum recruiting scenery of 70 patients were finally included in the present study from 16 January 2018 to 4 February 2020. The study was closed earlier than expected to achieve the maximum recruiting schedule (31 March 31 2020), due to the COVID-19 pandemic that strongly affected Spain. The number of recruited patients at that time was already over the expectation of the standard calculated sample size, heading for an alpha = 0.05 and beta = 0.2 error.

Four patients resulted in screening failure, and one patient retracted consent after the screening period. Patients and tumor characteristics for the 57 patients who started enzalutamide treatment are described in **Table 1**. Most patients (32/57, 73.7%) were classified as unfavorable intermediate NCCN risk subgroups.

**Table 1:** Patient Characteristics (n = 57).

	N
**Age (years)**	71.7 (50–83)
**T Stage**	
T1c	35 (61.4%)
T2a	15 (26.3%)
T2b	3 (5.3%)
T2c	4 (7.0%)
**N stage**	
Nx	0 (0%)
N0	57 (100%)
N1	0 (0%)
**M stage**	
Mx	0 (0%)
M0	57 (100%)
M1	0 (0%)
**Gleason Score**	
6	3 (5.3%)
7	54 (94.7%)
3 + 4	29 (53.7%)
4 + 3	25 (46.3%)
**Affected biopsy cores (%)**	38.7 (8–100)
**Pretreatment PSA ng/ml**	
≤10	46 (80.70%)
>10	11 (19.30%)
**NCCN risk subgroup**	
Favorable	15 (26.3%)
Unfavorable	32 (73.7%)
**ECOG**	
0	56 (98.24%)
1	1 (1.76%)
**Charlson score**	
0–1	48 (84.21%)
2	5 (8.77%)
3	3 (5.30%)
Unknown	1(1.76%)
**Body Mass Index**	
<25	8 (14.03%)
25–30	31 (54.38%)
>30	12 (21.05%)
Unknown	6 (10.52%)
**Basal Hypertension**	
<140/90	19 (33.33%)
>141/91	31 (54.38%)
Unknown	7 (12.28%)
**Basal elevated Cholesterol/Triglicerides**	
No	23 (40.35%)
Yes	19 (33.33%)
Unknown	15 (26.32%)
**Basal elevated ALT/AST**	
Yes	3 (5.27%)
No	54 (94.73%)

### Protocol Compliance and Security

**Figure 1:**
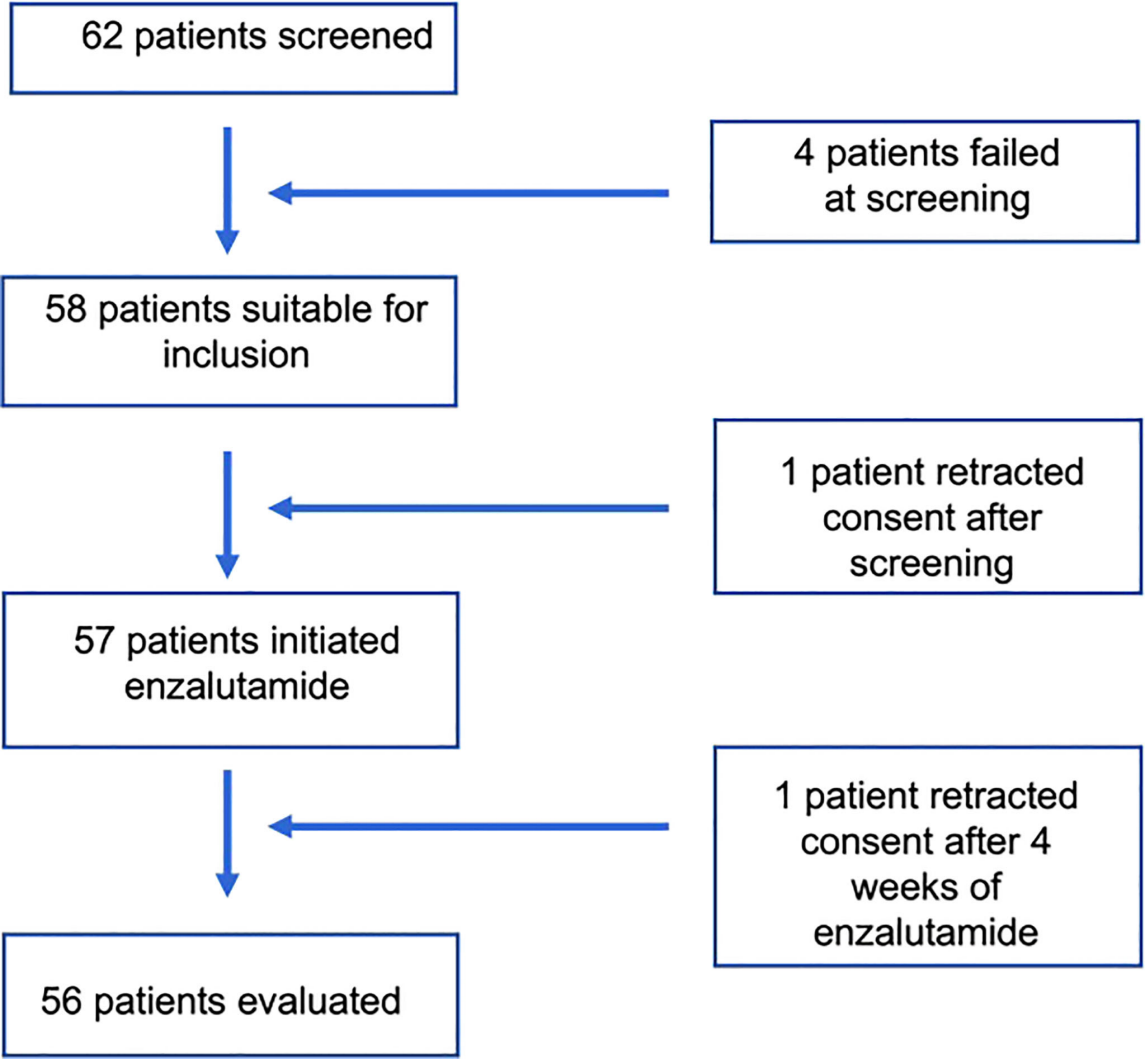
Protocol Flow Chart.

One of the 57 patients taking enzalutamide, retract consent to participate in the study at the 4th week due to general discomfort, unrelated to any objective toxicity. Therefore, 56 patients were finally included in the study (**Figure 1**).

During enzalutamide treatment, three severe adverse effects were reported. One severe hepatic toxicity (Grade 4) related to enzalutamide, displaying a rise in liver enzymes at the 7th week, normalized after complete and definitive enzalutamide cessation. The responsible investigator considered this adverse effect as related to enzalutamide. Anyhow, the patient continued with the study program evaluations and tests. Two patients suffered severe adverse effects non-related to enzalutamide. One patient had sepsis after fiducial implantation in the prostate for IGRT in the 2nd week, and one patient suffered an ictus in the 9th week. This patient had a previous hypertensive clinical history, and the event was not related by the responsible investigator to enzalutamide treatment. Both patients completed the enzalutamide treatment but with a dosage reduction to 120 mg/day as per protocol in the hypertensive patient.

One patient abandoned enzalutamide treatment at week 11 due to general discomfort unrelated to any objective toxicity. The patient agreed to continue the study follow-up. Two patients from the same center misunderstood the trial instructions and stopped enzalutamide during the 5 weeks of radiotherapy treatment.

Radiotherapy was administered as scheduled (total dose of 70 Gy in 28 fractions, 2.5 Gy per fraction) to all 56 patients. All 56 cases but one (a patient who started radiotherapy in the 5th week) started radiotherapy between the 8th and the 13th week as scheduled. Radiotherapy was completed in all cases, for a total treatment time of 41.63 ± 3.30 days (CI 95% 40.75–42.51). Dosimetry recommendations were well accomplished in all cases. IIn most cases, PTV coverage and OAR constraints were achieved in most cases (**Supplementary Material Table 2**).

Acute toxicity was recorded as the maximum toxicity observed during treatment and until one month after cessation of enzalutamide (**Table 2**). Two patients, as described above, presented grade 4 toxicity (hypertensive in one case, liver enzyme elevation in the other case). Severe grade 3 acute systemic toxicity observed was related to hypertension (systolic in all cases) in 19/56 (33.93%). Urinary and gastrointestinal toxicity 2 were present in 18/56 (32.14%) and 5/56 (8.9%) patients, respectively. Common (one third of the cases) mild toxicity included asthenia, breast pain, gynecomastia, urinary pain, and polaquiuria (**Table 3**). Other acute general, hormonally-related and gastrointestinal toxicity were also mild and uncommon.

**Table 2:** Maximum grade acute and late adverse effect after treatment in 56 evaluable patients.

	Acute Toxicity			Late Toxicity		
	G1	G2	G3	G1	G2	G3
**General Symptoms**						
Hypertension	10 (17.86%)	26 (46.43%)	19 (33.93%)	18 (45.0%)	17 (42.50%)	5 (12.50%)
Asthenia	18 (32.14%)	3 (5.36%)		2 (3.57%)		
AST/ALT elevation	11 (19.64%)					
Somnolence/Insomnia	5 (8.93%)			3 (5.36%)		
Headache/loss of concentration	1 (1.79%)	1 (1.79%)		1 (1.79%)		
Dizzines/ortostasim	6 (10.71%)	1 (1.79%)	1 (1.79%)		1 (1.79%)	
Depression/Anxiety	1 (1.79%)	1 (1.79%)		3 (5.36%)		
Dry skin	3 (5.36%)	1 (1.79%)		2 (3.57%)		
Skin hyperpigmentation folliculitis	2 (3.57%)			1 (1.79%)		
Mialgia/leg discomfort	3 (5.36%)					
Arthralgia	3 (5.36%)			1 (1.79%)		
**Symptoms related to hormonal changes**						
Breast Pain	14 (25.00%)	3 (5.36%)		11 (19.64%)		
Nipple pain/discomfort	3 (5.36%)			1 (1.79%)		
Gynecomastia	13 (23.21%)	5 (8.93%)		8 (14.29%)		
Hot flashes	2 (3.57%)			1 (1.79%)		
Libido Decreased	7 (12.50%)			6 (10.71%)		
Retrograde ejaculation	2 (3.57%)			2 (3.57%)		
Hipogonadism	2 (3.57%)			1 (1.79%)		
**Urinary symptoms**						
Pain	12 (21.43%)	6 (10.71%)	1 (1.79%)			1 (1.79%)
Urgency	13 (23.21%)	6 (10.71%)	3 (5.36%)	1 (1.79%)		
Incontinence	9 (16.07%)			1 (1.79%)		
Polaquiuria	4 (7.14%)	8 (14.81)	2 (3.57%)			
Retention/obstruction	2 (3.57%)	4 (7.14%)	1 (1.79%)			1 (1.79%)
Non infectous cystitis	4 (7.14%)	1 (1.79%)				
Nicturia		1 (1.79%)				
**Gastrointestinal Symptoms**						
Abdominal Pain	4 (7.14%)			2 (3.57%)		
Rectal Pain	4 (7.14%)	1 (1.79%)		1 (1.79%)		
Proctitis	7 (12.50%)	2 (3.57%)				1 (1.79%)
Anorexia/Hyporexia	5 (8.93%)	1 (1.79%)		1 (1.79%)		
Disgeusia	2 (3.57%)	1 (1.79%)				
Constipation/Diarrhea	8 (14.29%)					
Nausea/Vomitting	5 (8.93%)	1 (1.79%)				
Meteorism	2 (3.57%)					

Acute grade 4 was observed in 2 patients (one hypertensive crisis and one elevation of AST/ALT). No grade 4 late toxicity was observed.

**Table 3:** PSA decline values at pre-specified time points.

	25th week (n = 50)	1 month after enzalutamide (n = 51)	3 months after enzalutamide (n = 51)	6 months after enzalutamide (n = 51)
PSA decline ≥80%	50/50 (100%)(95% CI: 92.89–100%)	51/51 (100%)(95% CI: 93.02–100%)	51/51 (100%)(95% CI: 93.02–100%)	51/51 (100%)(95% CI: 93.02–100)
PSA decline ≥90%	50/50 (100%)(95% CI: 92.89–100%)	51/51 (100%)(95% CI: 93.02–100%)	46/51 (90.2%)(95% CI: 78.59–96.74%)	45/51 (88,24%)(95% CI: 76.13–95.56%)
PSA <0.2 ng/ml	50/50 (100%)(95% CI: 92.89–100%)	42/51 (82.3%)(95% CI: 69.13–91.6%)	29/51 (56.8%)(95% CI: 42.25–70.65%)	26/51 (50.98%)(95% CI: 36.6–65.25%)
PSA <0.1 ng/ml	44/50 (88%)(95% CI: 75.69–95.47%)	37/51 (72.5%)(95% CI: 58.26–84.11%)	13/51 (25.5%)(95% CI: 14.33–39.63)	9/51 (17.6%)(95% CI: 8.4–30.87%)

Late toxicity was recorded 6 months after enzalutamide cessation. Most of the urinary and hypertensive severe toxicity disappeared. Toxicity was mainly related to hormonally derived symptoms such as breast pain and gynecomastia. Severe grade 3 toxicity was present in 2 patients, one with urinary pain and retention, and the other showing grade 3 proctitis. Grade 3 hypertension was observed in 5 patients (**Table 2**).

### PSA

All 56 patients included in the study were analyzed for PSA response in an intention-to-treat analysis and evaluated according to the PSA response data time-point available. All 56 patients evaluable for PSA treatment-induced modifications at pre-specified time points showed PSA reduction higher than 80%. At the 25th week, all evaluable patients (50 cases) achieved PSA values of 0.2 ng/ml and PSA was under detectable levels (<0.1 ng/ml) in 92% of all patients (**Table 3**). PSA values dropped from pretreatment levels of 7.61 ± 2.82 (3.53–16.77) ng/ml to 0.04 ± 0.04 (0.00–0.16) ng/ml at the 25th week and remained low 6 months after cessation of enzalutamide (**Table 4**).

**Table 4:** PSA and hormone profile values at pre-specified time points.

	Pretreatment	4th week	25th week	1 monthafter enza	3 months after enza	6 months after enza	P-value
**PSA (**ng/ml)	(n = 56)	(n = 52)	(n = 50)	(n = 51)	(n = 51)	(n = 51)	Pre vs 4th w: p <0.0001 Pre vs 25th w p <0.0001
Mean ± SD	7.61 ± 2.82	2.98 ± 2.37	0.04 ± 0.04	0.09 ± 0.12	0.28 ± 0.29	0.29 ± 0.28	Pre vs 1 m p <0.0001
(range)	(3.53–16.77)	(0.22–11.50)	(0.00–0.16)	(0.00–0.52)	(0.01–1.21)	(0.01–1.11)	Pre vs 3 m p <0.0001
95% CI	6.87–8.35	2.33–3.62	0.03–0.05	0.06–0.12	0.20–0.36	0.21–0.36	Pre vs 6 m p <0.0001
**Testosterone (**ng/ml)	(n = 53)	(n = 48)	(n = 48)	(n = 48)	(n = 49)	(n = 46)	Pre vs 4th w: p <0.0001 Pre vs 25th w p <0.0001
Mean ± SD	5.41 ± 2.74	9.83 ± 4.18	9.16 ± 4.52	8.04 ± 4.25	7.49 ± 10.55	4.82 ± 3.633	Pre vs 1 m p <0.0001
(range)	(2.20–18.19)	(3.11–19.00)	(1.70–21.10)	(1.40–24.27)	(1.70–69.35)	(1.30–25.91)	Pre vs 3 m p = 0.154
95% CI	4.68–6.15	8.65–11.02	7.88–10.44	6.84–9.25	4.53–10.44	3.77–5.87	Pre vs 6 m p = 0.285
**Estradiol (**pg/ml)	(n = 48)	(n = 44)	(n = 45)	(n = 44)	(n = 41)	(n = 39)	Pre vs 4th w: p <0.0001 Pre vs 25th w p <0.0001
Mean ± SD	26.52 ± 9.59	44.40 ± 17.56	41.72 ± 19.93	40.37 ± 16.78	30.05 ± 10.61	30.59 ± 10.62	Pre vs 1 m p <0.0001
(range)	(10.00–47.70)	(14.00–87.00)	(0.00–85.00)	(0.00–72.00)	(12.00–51.00)	(10.00–54.00)	Pre vs 3 m p = 0.015
95% CI	23.80–29.23	39.21–49.59	35.90–47.54	35.41–45.33	26.80–33.30	27.25–33.92	Pre vs 6 m p = 0.057
**LH (**mUl/ml)	(n = 51)	(n = 49)	(n = 49)	(n = 46)	(n = 44)	(n = 45)	Pre vs 4th w: p <0.0001 Pre vs 25th w p <0.0001
Mean ± SD	6.99 ± 4.98	13.19 ± 6.69	19.24 ± 8.46	17.49 ± 8.47	12.56 ± 6.67	11.12 ± 5.43	Pre vs 1 m p <0.0001
(range)	(2.08–30.72)	(4.83–35.95)	(7.34–39.20)	(7.24–39.40)	(5.50–32.60)	(4.68–30.24)	Pre vs 3m p <0.0001
95% CI	5.62–8.36	11.32–15.07	16.87–21.61	15.04–19.94	10.59–14.53	9.54–12.71	Pre vs 6m p <0.0001
**FSH (**mUl/ml)	(n = 48)	(n = 45)	(n = 44)	(n = 41)	(n = 40)	(n = 40)	Pre vs 4th w: p = 0.055 Pre vs 25th w p <0.0001
Mean ± SD	13.73 ± 18.77	15.11 ± 18.68	28.86 ± 15.51	27.14 ± 13.95	27.49 ± 12.08	27.43 ± 12.70	Pre vs 1 m p <0.0001
(range)	(2.20–126.24)	(2.30–116.44)	(9.20–81.17	(11.40–88.66)	(10.68–79.05)	(9.51–84.71)	Pre vs 3m p <0.0001
95% CI	8.42–19.04	9.66–20.57	24.27–33.44	22.87–31.41	23.74–31.23	23.49–31.36	Pre vs 6m p <0.0001

### Hormone Levels

**Figure 2:**
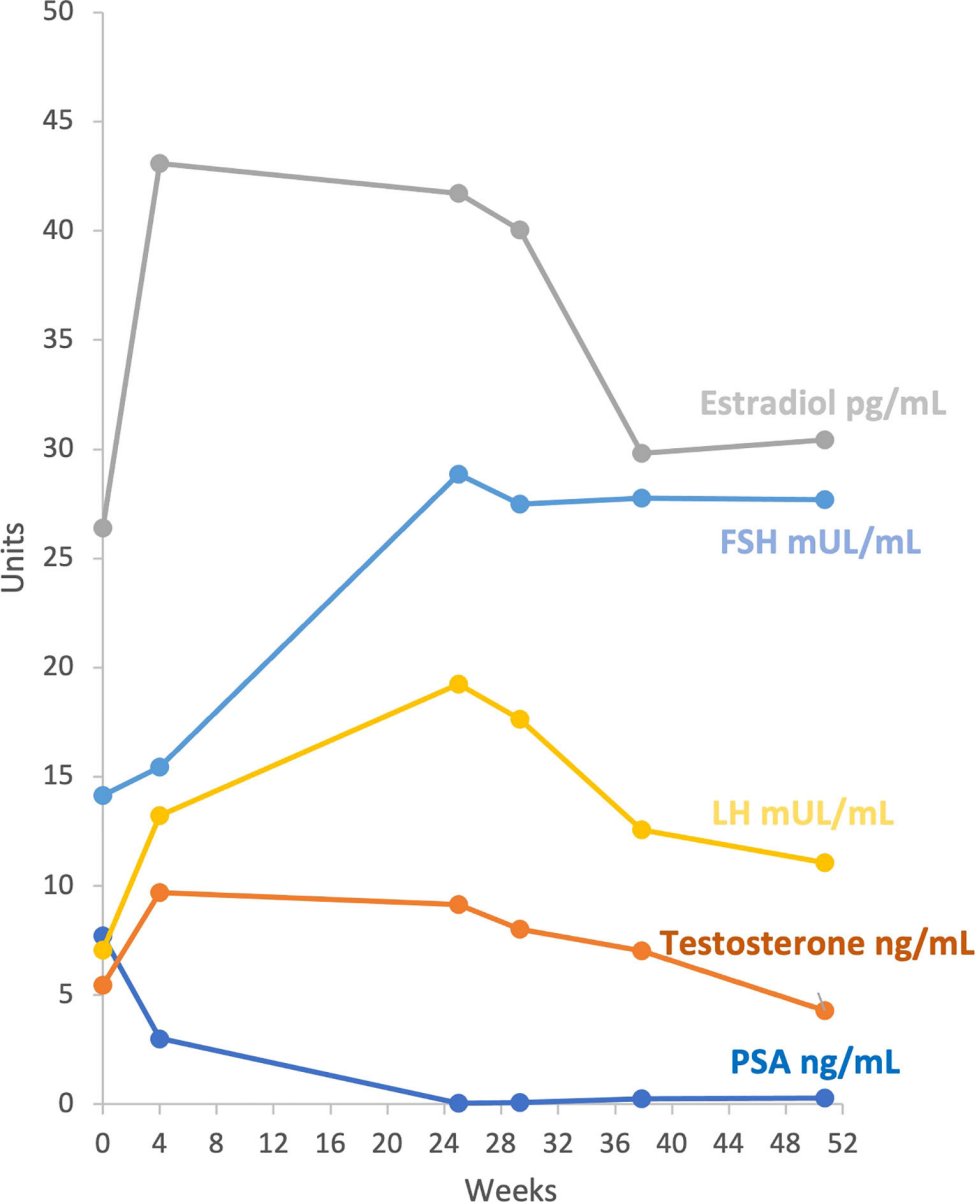
Graphical presentation of PSA and Hormonal profile at pre-specified timepoints. Data in Y-axis represent number of units (type of units for each parameter is displayed in the figure lines). Data in X- axis represent number of weeks after pretreatment assessment.

Patients treated with enzalutamide showed a sharp increase in testosterone and estradiol after 4 weeks of enzalutamide treatment (**Table 4**). LH and FSH levels were also increased at week 25. Testosterone and estradiol levels decreased to pretreatment levels, but LH and FSH levels remained elevated at 6 months (**Figure 2**).

Anthropometric, bone, and metabolic changes at a pre-specified time point.

At the time of last evaluation, there was no statistically significant weight change after enzalutamide treatment, either in bone density as measured in densitometric analysis or the bone resorption marker, alkaline phosphatase. Metabolic changes in fasting glucose, cholesterol, or triglyceride levels were not present after enzalutamide treatment. There was a modest increase in HDL cholesterol at the last evaluation (**Table 5**).

**Table 5:** Antropometric, bone and metabolic changes at pre-specified time point.

	Pretreatment	12th week	25th week	1 month after enza	P-value
Body Mass Index	(n = 50)	(n = 46)	(n = 40)	(n = 40)	
Mean ± SD	28.30 ± 4.55	27.55 ± 4.83	27.30 ± 4.18	27.38 ± 4.11	Pre vs 12th w: p <0.0001
(range)	(17.03–44.39)	(16.78–44.46)	(17.32–40.04)	(20.57–40.57)	Pre vs 25thw: p = 0.001
95% CI	27.04–29.56	26.16–28.95	26.01–28.60	26.11–28.66	Pre vs 1 m: p = 0.082
**Bone Density Femoral Neck (g/cm^2^)**	(n = 45)		(n = 45)		
Mean ± SD	0.85 ± 0.15		0.86 ± 0.15		Pre vs 25th w: p = 0.253
(range)	(0.58–1.35)		(0.61–1.25)		
95% CI	0.80–0.89		0.82–0.91		
**Bone Density Lumbar Spine (g/cm^2^)**	(n = 48)		(n = 48)		
Mean ± SD	1.13 ± 0.21		1.14 ± 0.21		Pre vs 25^th^ w: p = 0.342
(range)	(0.77–1.87)		(0.77–1.92)		
95% CI	1.07–1.19		1.08–1.20		
**Alkaline Phosphatase (U/L)**	(n = 49)	(n = 44)	(n = 49)		
Mean ± SD	72.61 ± 27.65	65.48 ± 17.63	76.59 ± 25.78		Pre vs 12thw: p = 0.033 Pre vs 25thw: p = 0.093
(range)	(39.00–226.00)	(38.00–139.00)	(36.00–186.00)		
95% CI	64.87–80.36	60.27–70.69	69.37–83.81		
**Fasting Glucose (mg/dl)**	(n = 54)	(n = 46)	(n = 50)	(n = 50)	
Mean ± SD	113.67 ± 31.71	113.43 ± 28.76	115.68 ± 31.95	117.94 ± 32.22	Pre vs 12thw: p = 0.881
(range)	(83.00–253.00)	(70.00–253.00)	(83.00–247.00)	(80.00–263.00)	Pre vs 25thw: p = 0.886
95% CI	105.21–122.12	105.12–121.75	106.82–124.53	108.01–125.87	Pre vs 1 m: p = 0.758
**Fasting Cholesterol Total (mg/dl)**	(n = 38)	(n = 24)	(n = 37)		
Mean ± SD	185.58 ± 40.56	189.88 ± 34.69	198.44 ± 38.42		Pre vs 12thw: p = 0.218
(range)	(114.00–277.00)	(117.00–254.00)	(102.00–269.00)		Pre vs 25thw: p = 0.054
95% CI	172.69–198.48	176.00–203.75	186.06–210.82		
**Fasting Cholesterol HDL (mg/dl)**	(n = 33)	(n = 21)	(n = 32)		
Mean ± SD	54.19 ± 22.46	49.72 ± 10.30	58.37 ± 22.12		Pre vs 12thw: p = 0.382
(range)	(34.00–162.00)	(34.00–69.00)	(41.00–162.00)		Pre vs 25thw: p = 0.015
95% CI	46.52–61.85	45.31–54.12	50.71–66.03		
**Fasting Cholesterol LDL (mg/dl)**	(n=33)	(n = 20)	(n = 32)		
Mean ± SD	109.34±40.64	108.08 ± 30,33	116.37 ± 30.64		Pre vs 12thw: p = 0.409
(range)	(16.00-197.00)	(57.00–173.00)	(70.00–174.00)		Pre vs 25thw: p = 0.220
95% CI	95.47-123.20	94.79–121.37	105.75–126.98		
**Fasting Triglicerides (mg/dl)**	(n = 38)	(n = 20)	(n = 38)		
Mean ± SD	130.22 ± 55.97	142.78 ± 61.16	136.85 ± 56.90		Pre vs 12thw: p = 0.971
(range)	(54.00–265.00)	(54.00–313.00)	(56.00–301.00)		Pre vs 25thw: p = 0.165
95% CI	112.42–148.02	119.71–165.85	118.49–154.68		

### Patients Reported Outcomes (PROs) at Pre-Specified Time Points

**Figure 3:**
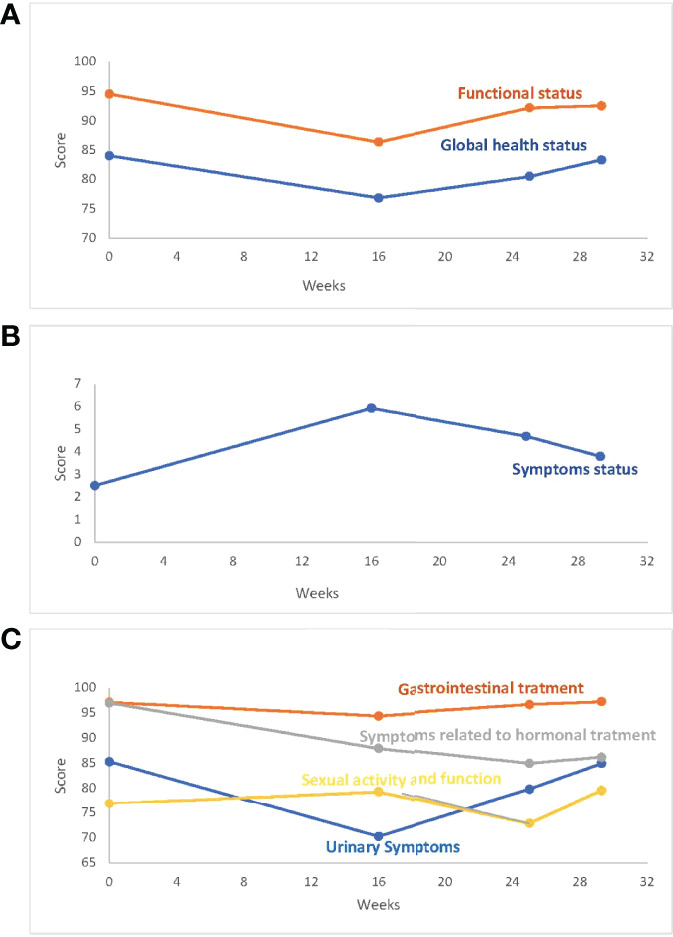
PRO **(A)**. QLQ30_Global Health Status/Functional Status. **(B)** QLQ30_Symptoms Status. **(C)** QLQ_PR25. PROs score is displayed in the Y-axis. Weeks after pretreatment assessment are displayed in the X-axis.

PROs were analyzed through the EORTC QLQC30 and EORTC QLQ-PR25 at pretreatment, the 12th week of treatment, at the 25th week, and one month after cessation of enzalutamide. A reduction in QoL scores as estimated by the EORTC QLQC30 and an increase in symptoms were observed at the 12th and 25th weeks, recovering one month after cessation of the treatment. Specific PRO analysis of symptoms related to prostate cancer treatment (EORTC-QLQ-PR25) showed a significant impact on the urinary domain during the radiotherapy treatment period (12th–25th week) that recovered one month after cessation of treatment. Gastrointestinal and sexual domains did not change significantly during treatment and completely recovered at the end of the study period. Changes in the hormonal domain remained significantly present one month after enzalutamide treatment (**Table 6**, **Figure 3**).

**Table 6:** Quality of Life assessment at pre-specified time points.

QLQ30	Pretreatment (n = 53)	12 th week (n = 50)	25th week (n = 47)	1 month after enzalutamide (n = 45)	P-value
Global Health					
Mean ± SD	82.55 ± 16.69	74.50 ± 19.30	80.50 ± 15.57	81.48 ± 18.02	Pre vs 12th w: p = 0.011
(range)	(33.33–100.00)	(16.67–100.00)	(50.00–100.00)	(33.33–100.00)	Pre vs 25th w: p = 0.155
95% **CI**	78.05–87.04	69.15–79.85	76.05–84.95	76.22–86.75	Pre vs 1 month: p = 0.682
**Functioning area**					
Mean ± SD	94.12 ± 7.19	88.53 ± 14.00	92.17 ± 8.53	92.05 ± 10.66	Pre vs 12th w: p = 0.011
(range)	(62.22–100.00)	(31.11–100.00)	(71.11–100.00)	(57.78–100.00)	Pre vs 25th w: p = 0.075
95% **CI**	92.18–96.05	84.65–92.41	89.73–94.61	88.97–95.13	Pre vs 1 month: p = 0.260
**Symptoms Area**					
Mean ± SD	2.95 ± 3.17	6.49 ± 6.45	4.71 ± 4.37	4.37 ± 4.75	Pre vs 12th w: p <0.001
(range)	(0.00–11.54)	(0.00–26.92)	(0.00–20.51)	(0.00–16.67)	Pre vs 25th w: p = 0.005
95% **CI**	2.10–3.80	4.70–8.28	3.46–5.96	3.00–5.75	Pre vs 1 month: p = 0.088
**QLQ-PR25**	**Pretreatment (n = 53)**	**12th week (n = 50)**	**25th week (n = 47)**	**1 month after enzalutamide (n = 47)**	**P-value**
**Urinary Symptoms**					
Mean ± SD	84.04 ± 12.60	69.51 ± 21.86	79.61 ± 17.64	81.84 ± 19.57	Pre vs 12th w: p <0.0001
(range)	(37.50–100.00)	(0.00–100.00)	(25.00–100.00)	(16.67–100.00)	Pre vs 25th w: p = 0.031
95% **CI**	80.65–87.43	63.45–75.57	75.57–84.65	76.39–87.29	Pre vs 1 month: p = 0.470
**Gastrointestinal Symptoms**					
Mean ± SD	97.01 ± 5.91	94.33 ± 9.44	96.45 ± 6.89	96.92 ± 5.92	Pre vs 12th w: p = 0.059
(range)	(75.00–100.00)	(50.00–100.00)	(75.00–100.00)	(75.00–100.00)	Pre vs 25th w: p = 0.569
95% **CI**	95.42–98.60	91.72–96.95	94.48–98.43	95.21–98.63	Pre vs 1 month: p = 0.844
**Hormonal related symptoms**					
Mean ± SD	96.54 ± 5.99	87.22 ± 10.86	84.75 ± 12.40	85.93 ± 10.42	Pre vs 12th w: p <0.0001
(range)	(72.22–100.00)	(50.00–100.00)	(55.56–100.00)	(55.56–100.00)	Pre vs 25th w: p <0.0001
95% **CI**	94.93–98.15	84.21–90.23	81.18–88.33	82.96–88.91	Pre vs 1 month: p <0.0001
**Sexual activity**					
Mean ± SD	77.04 ± 19.97	80.79 ± 25.00	72.86 ± 25.84	78.70 ± 25.84	Pre vs 12th w: p = 0.550
(range)	(33.33–100.00)	(5.56–100.00)	(11.11–100.00)	(16.67–100.00)	Pre vs 25th w: p = 0.232
95% **CI**	71.67–82.42	73.71–87.86	65.47–80.25	71.86–85.54	Pre vs 1 month: p = 0.822

## Discussion

Patients having localized intermediate prostate cancer are usually treated with a combination of radiation therapy and 6 months of ADT. Previous studies have shown an excellent toxicity profile of enzalutamide monotherapy compared with ADT (26). Furthermore, combined enzalutamide and conventionally fractionated radiotherapy has been shown to be well tolerated in this particular clinical situation (38). But little is known about the toxicity and PROs when enzalutamide monotherapy is discontinued.

Our study was planned to assess the role of enzalutamide monotherapy combined with modern hypofractionated EBRT for treating patients with localized intermediate-risk prostate cancer.

As previously described in other studies (24), our patients showed a better toxicity profile than that traditionally described in ADT trials, caused by the compensatory elevation of sexual hormones. No changes in body mass index, bone density mass, fasting glucose, cholesterol, or libido were found one month after the end of enzalutamide. Just after the end of enzalutamide treatment, modest changes in HDL-cholesterol were still evident.

As expected, testosterone, estradiol, LH, and FSH levels sharply increase during enzalutamide treatment (24). Our data showed for the first time that testosterone and estradiol levels tend to return to basal levels 6 months after cessation of enzalutamide, although LH and FSH remain elevated.

This fact, would be relevant when assessing the acute and long-term hormonal side effects analyzed either by the physicians, through the CTCAE4.0 toxicity scale [Physician Reported Outcomes, (PhyROs)] or the patients, through the EORTC QLQC30 and EORTC QLQ-PR25 (PROs). In fact, no sexual toxicity was observed, but gynecomastia (CTCAE 4.0) and hormonal related symptoms (QLQPR25) remained a problem for patients one month after the end of enzalutamide treatment.

In contrast, the global health status, the functioning area, or symptoms other than hormonally related, returned to pretreatment levels one month after cessation of enzalutamide.

The use of Hypo-EBRT is also a novelty in our study. We treated our patients according to the Hypo-EBRT protocol described by Kupelian et al. (34) and as the treatment arm in the RTOG 0415 trial (6). This schedule and others (39) provide the highest EQD2 (80 Gy) to the PTV, compared to other hypofractionated schemes (5–7). Our acute GU toxicity was slightly higher than that observed in the hypofractionated arm of the RTOG 0415 (32.9% vs 27%), while GI toxicity was very similar (8.9% vs 10.7%). Our 80 Gy EQD2 PTV included the proximal seminal vesicles (the first 1 cm of the seminal vesicles). This extra volume was not treated in the RTOG trial, as only low-risk patients were included in that trial. This higher PTV volume would be related to the slightly increased urinary toxicity found in our study (6). In the RTOG 0415 study, the hypofractionated arm had a very similar toxicity profile to the conventional arm. This conventionally fractionated radiotherapy scheme had a lower EQD2 (70 Gy) (6).

The study from Kaplan et al. (38) already analyzed this possibility by combining standard escalated cEBRT with enzalutamide monotherapy in intermediate-risk prostate cancer patients. Patients received conventionally fractionated EBRT to a total dose of 79.2 Gy at 1.8 Gy per fraction for 44 fractions (9 weeks), and enzalutamide was administered for 6 months. They reported only 6 cases out of 45 (13.33%) of ≧grade 2 urinary frequency. We observed this particular toxicity in 9/56 patients (16.06%). No data are available regarding the other GU toxicity items described in our study. We must note that due to the selected radiotherapy treatment in the Kaplan study (38) (1.8 Gy per fraction, 44 fractions to a total dose of 79.2), the EQD2 of this cEBRT is 74.67 Gy. This equivalent dose is well below the 80 Gy administered in our study.

The PROs recognized a temporary increase in urinary scores in the evaluations performed in the 12th week (just after the end of Hypo-EBRT) that was rapidly recovered at the end of the study period. However, no gastrointestinal or sexual symptom scores were changed.

The primary endpoint of the study deals with the efficacy of the combination of enzalutamide monotherapy and modern Hypo-EBRT, in terms of reduction of PSA levels, in patients with localized intermediate-risk prostate cancer, as used in similar trials (24–26, 38).

As stated earlier, activity analysis was performed with the intention of treating conditions. The PSA response was analyzed by the proportion of patients who showed a reduction of at least 80% of the initial values at the end of the 25 weeks of enzalutamide treatment. The seminal study by Tombal et al. (24) showed a PSA response of 92.5% (95% CI 86.2–98.8), similar to the 100% observed in our study. We also analyzed the kinetics of PSA reduction at pre-specified time-points (1, 3, and 6 months) after the cessation of enzalutamide. Our study showed that all patients remain in PSA response 6 months after the cessation of enzalutamide. Furthermore, 90% of the patients still showed a PSA decline of 90% of the pretreatment values, 6 months after the enzalutamide cessation. Obviously, the effect of radiotherapy on this maintained PSA decline is to be taken into account.

The study by Kaplan et al. (38) combined conventionally fractionated radiotherapy with enzalutamide monotherapy in intermediate-risk prostate cancer. They defined PSA response as PSA levels lower than 0.2 ng/ml at the end of 25 weeks of enzalutamide (39). Forty-nine out of 62 (79%) of their patients showed PSA response, in compared with 51/51 (100%) in our series for the same response evaluator. No data on PSA response was given after enzalutamide cessation in the Kaplan study, but 56.8% of our patients remained in the PSA response (<0.2 ng/ml) 6 months after enzalutamide cessation. Again, the lower EQD2 radiation dose in this study (74.67 Gy) the the present one (80 Gy) would explain the lower response rate observed.

The effect of radiotherapy along with enzalutamide versus enzalutamide alone, would only be indirectly analyzed by comparing the results from Tombal et al. (24) with those results from Kaplan and this study. Enzalutamide alone provided a 45% rate of undetectable PSA (<0.1 ng/ml) compared with 61.3% (38/62) for cEBRT and 88% for Hypo-EBRT. Although patient and tumor characteristics are of poorer prognosis in the enzalutamide alone trial (24), these data would shed light on the effect of radiotherapy along with enzalutamide in this particular setting.

Although available results regarding the role of enzalutamide and hypofractionated radiotherapy (38 and present series) are limited by the short follow-up, recent evidence seems to confirm the role of this approach in prostate cancer patients. Long-term evidence for the role of antiandrogen monotherapy as an alternative to ADT combined with hypofractionated radiotherapy comes from the CHiiP trial (40). In a *post hoc* analysis, they compared the results of 2,700 patients who received LHRHa and those of 403 patients who received bicalutamide (150 mg/day) as concomitant hormonal treatment. All characteristics of patient and tumor were similar among the two groups unless bicalutamide patients were significantly younger (median 67 vs 69 years LHRHa). After a median follow-up of 9.3 years, there was no difference in biochemical or clinical failure. Late toxicity, as estimated by the LENT-SOMA, was more frequently reported in LHRHa patients compared to bicalutamide patients. The quality of life was similar in both arms.

These mature results of a first-generation antiandrogen (bicalutamide) in monotherapy combined with hypofractionated radiotherapy would probably be confirmed when using a more active second-generation antiandrogen like enzalutamide in a similar setting.

The improvement in PSA response by adding radiotherapy to enzalutamide and the better response observed when using modern hypofractionated EBRT are related, in our opinion, not only to the higher EQD2 administered but to the biological basis of the radiosensitizing effect of enzalutamide. If protracted conventional radiotherapy schemes are used (daily fractions for almost nine weeks), tumor proliferation would be relevant during radiotherapy, achieving tumor repopulation during this very long treatment time and therefore, reducing tumor control induced by radiation (41). Furthermore, conventionally fractionated radiotherapy probably does not take full advantage of the increased radiosensitation observed when enzalutamide is given in the presence of fractions higher than 2 Gy (hypofractionated radiotherapy) (38).

We can conclude that the treatment schedule proposed here for the first time is safe and very active in reducing the PSA levels. Our study also showed that such a PSA reduction is maintained 6 months after the cessation of enzalutamide treatment. Longer follow-up is needed to confirm the potential use of this combination in future randomized trials.

## Data Availability Statement

The data presented in the study is available on request, further inquiries can be directed to the corresponding author/s.

## Ethics Statement

The studies involving human participants were reviewed and approved by the CEIm University Hospital Canarias. The patients/participants provided their written informed consent to participate in this study.

## Author Contributions

All authors listed have made a substantial, direct, and intellectual contribution to the work and approved it for publication.

## Funding

This is an independent academic study (Canarian Foundation of Health Investigations, FUNCANIS) supported by an unrestricted educational grant from Astellas.

## Conflict of Interest

PL received payment or honoraria for lectures, presentations, speakers bureaus, manuscript writing or educational events from Carl-Zeiss and support for attending meetings and/or travel from Carl-Zeiss. JR-M received payment or honoraria for lectures, presentations, speakers bureaus, manuscript writing or educational events from ASTELLAS, BAYER, JANSSEN, CASEN-RECORDATI, IPSEN PHARMA & BOSTON SCIENTIFIC and support for attending meetings and/or travel from ASTELLAS & JANSSEN. He also received equipment, materials, drugs, medical writing, gifts or other services from ASTELLAS, BAYER, JANSSEN, CASEN-RECORDATI, IPSEN PHARMA & BOSTON SCIENTIFIC. AP-E received payment or honoraria for lectures, presentations, speakers bureaus, manuscript writing or educational events from ASTELLAS, JANSSEN & IPSEN PHARMA and support for attending meetings and/or travel from ASTELLAS & JANSSEN. AL declared payment or honoraria for lectures, presentations, speakers bureaus, manuscript writing or educational events from ASTELLAS, JANSSEN & IPSEN PHARMA and declared participation on a Data Safety Monitoring Board or Advisory Board JANSSEN & ASTELLAS. EV received payment or honoraria for lectures, presentations, speakers bureaus, manuscript writing or educational events from JANSSEN &IPSEN PHARMA, support for attending meetings and/or travel from ELEKTA, and declared participation on a Data Safety Monitoring Board or Advisory Board for JANSSEN. She is also the National Coordinator of the Brachytherapy Group of the Spanish Society of Radiation Oncology (SEOR). AG-I received payment or honoraria for lectures, presentations, speakers bureaus, manuscript writing, or educational events from ASTELLAS, BAYER, JANSSEN, IPSEN PHARMA & BOSTON SCIENTIFIC and declared participation on a Data Safety Monitoring Board or Advisory Board for BAYER, ELEKTA, JANSSEN & ASTELLAS. GS received payment or honoraria for lectures, presentations, speakers bureaus, manuscript writing, or educational events from ASTELLAS, BAYER & JANSSEN and support for attending meetings and/or travel from ASTELLAS & IPSEN PHARMA. XM received payment or honoraria for lectures, presentations, speakers bureaus, manuscript writing, or educational events from ASTELLAS & BAYER and support for attending meetings and/or travel from ASTELLAS, IPSEN PHARMA & JANSSEN. He declared Participation on a Data Safety Monitoring Board or Advisory Board for BAYER &ASTELLAS. The remaining author declares that the research was conducted in the absence of any commercial or financial relationships that could be construed as a potential conflict of interest.

## Publisher’s Note

All claims expressed in this article are solely those of the authors and do not necessarily represent those of their affiliated organizations, or those of the publisher, the editors and the reviewers. Any product that may be evaluated in this article, or claim that may be made by its manufacturer, is not guaranteed or endorsed by the publisher.
