# Supplementary material for: Phase II Study of ENZAlutamide Combined With Hypofractionated Radiation Therapy (ENZART) for Localized Intermediate Risk Prostate Cancer
Source: Front Oncol. 2022 Jul 14;12:891886. doi: 10.3389/fonc.2022.891886 (PMC9329530; doi:10.3389/fonc.2022.891886)
Supplement: Supplementary file 1 [file Table_1.docx]

Supplementary Material Table 1.- Inclusion and exclusion criteria

Inclusion Criteria:

- Life expectancy of greater than 1 year.
- Diagnosis of histologically confirmed prostate carcinoma
- ECOG Score ≤ 1
- Participants must have an adequate organic function, defined as:
  1. Leukocytes ≥3,000/mcL
  2. Platelets ≥80,000/mcL
  3. Total bilirubin < 2X institutional upper limit
  4. AST (SGOT)/ALT (SGPT) ≤ 2.5 X institutional upper limit of normal
  5. Creatinine < 2x institutional limits .
- Potentially fertile patients should use effective contraceptive methods (barrier methods plus other contraceptive methods) before entering the study and during their participation in the study
- Ability to understand and the willingness to sign a written informed consent
- Patients should be available for clinical follow-up.
- To be able to swallow the medication of the study and to fulfill the requirements of the same one

Exclusion Criteria:

- Received an investigational agent within 4 weeks prior to enrollment
- Stage T4 prostate cancer by clinical examination or radiologic evaluation.
- Hypogonadism or severe androgen deficiency as defined by screening serum testosterone less than 50 ng/dL below the normal range for the institution.
- Prior androgen deprivation, chemotherapy, surgery, or radiation for prostate cancer.
- Receiving concurrent androgens, anti-androgens, estrogens, or progestational agents, or received any of these agents within the 6 months prior to enrollment or having taken finasteride or dutasteride within 30 days of registration.
- History of another active malignancy within the previous 5 years other than curatively treated nonmelanomatous skin cancer and superficial bladder cancer. Participants treated for malignancy with no relapse within two years are eligible to participate in the study.
- Uncontrolled intercurrent illness including, but not limited to, ongoing or active infection. Severe concurrent disease, infection, or co-morbidity that, in the judgment of the Investigator, would make the patient inappropriate for enrollment.
- History of seizure or any condition or concurrent medication that may predispose to seizure.
- History of loss of consciousness or transient ischemic attack within 12 months prior to enrollment.
- Clinically significant cardiovascular disease, including:
  1. Myocardial infarction within 6 months of enrollment
  2. Uncontrolled angina within 3 months of enrollment
  3. Congestive heart failure New York Heart Association (NYHA) class 3 or 4, or history of congestive heart failure NYHA class 3 or 4 in the past, unless a screening echocardiogram or multi-gated acquisition scan performed within 3 months results in a left ventricular ejection fraction ≥ 45%;
  4. History of clinically significant ventricular arrhythmias (e.g., ventricular tachycardia, ventricular fibrillation, torsades de pointes);
  5. History of Mobitz II second degree or third degree heart block without a permanent pacemaker in place;
  6. Hypotension as indicated by systolic blood pressure < 86 mmHg on 2 consecutive measurements at the Screening visit;
  7. Bradycardia as indicated by a heart rate < 50 beats per minute at the Screening visit;
  8. Uncontrolled hypertension as indicated by systolic blood pressure > 170 mmHg or diastolic blood pressure > 105 mmHg on 2 consecutive measurements at the screening visit;
  9. EKG demonstrating equal to or greater than grade III toxicity according the NCI Common Terminology Criteria for Adverse Events (CTCAE) version 4.0
- History of gastrointestinal disorders (medical disorders or extensive surgery) that may interfere with the absorption of oral study drug(s) within 3 months of enrollment.
- Major surgery within 4 weeks of registration.
- Previous use, or participation in a clinical trial, of an investigational agent that blocks androgen synthesis (e.g., abiraterone acetate, TAK-700, TAK-683, TAK-448) or targets the androgen receptor (e.g., enzalutamide, BMS 641988); ketoconazole.
- Any condition or reason that, in the opinion of the Investigator, interferes with the ability of the patient to participate in the trial, places the patient at undue risk, or complicates the interpretation of safety data.
- Use of herbal or alternative remedies that may affect hormonal status such as Prostasol or PC-SPES.
