# Supplementary material for: Phase II Study of ENZAlutamide Combined With Hypofractionated Radiation Therapy (ENZART) for Localized Intermediate Risk Prostate Cancer
Source: Front Oncol. 2022 Jul 14;12:891886. doi: 10.3389/fonc.2022.891886 (PMC9329530; doi:10.3389/fonc.2022.891886)
Supplement: Supplementary file 2 [file Table_2.docx]

Supplementary Material Table 2.- Data on PTV (A) and OAR, Rectum (B), Bladder (C), Right Femur (D), Left Femur(E) and Penis Bulb (F), Constraints as per protocol.

| **(A)**  **PTV** | **Volume (cc)**  (n=55) | **V100 (%)**  (n=56) | **% Variation Max.Dose**  (n=56) |
| --- | --- | --- | --- |
| **Patients out of constraint** |  | 10 | 0 |
| **Mean±SD (range)**  **IC95%** | 140.49±59.09  (6.78-293.83)  124.87-152.10 | 97.29±3.47  (88.00-103.00)  96.38-98.20 | 105.40±1.11  (103.80—109.34)  105.11-105.69 |

| **(B)**  **Rectum** | **Volume (cc)**  (n=56) | **Máx.Dose (Gy)**  (n=56) | **% Variation Max.Dose**  (n=56) | **V65 (%)**  (n=56) | **V60 (%)**  (n=56) | **V55 (%)**  (n=56) | **V40 (%)**  (n=56) |
| --- | --- | --- | --- | --- | --- | --- | --- |
| **Patients out of constraint** |  | 44 | 1 | 3 | 1 | 0 | 0 |
| Mean±SD  (range)  IC95% | 66.00±20.66  (25.20-131.70)  60.59-71.41 | 70.64±4.64  (39.00-75.00)  69.43-71.86 | 0.92±6.63  (-44.29-7.14)  -0.82-2.66 | 6.67±5.11  (0.00-35.00)  5.33-8.01 | 9.59±5.77  (0.00-40.00)  8.08-11.11 | 14.03±7.60  (1.70-45.00)  12.04-16.02 | 30.43±11.90  (4.68-52.00)  27.31-33.55 |

| **(C)**  **Bladder** | **Volume (cc)**  (n=54) | **Max Dose (Gy)**  (n=52) | **% Variation**  **Max.Dose**  (n=52) | **V65 (%)**  (n=54) | **V56 (%)**  (n=52) | **V52 (%)**  (n=54) |
| --- | --- | --- | --- | --- | --- | --- |
| **Patients out of constraint** |  | 48 | 8 | 0 | 0 | 0 |
| **Mean±SD**  **(range)**  **IC95%** | 272.82±177.76  (71.76-1161.70)  226.26-319.37 | 71.79±3.84  (46.00-75.67)  70.78-72.79 | 2.55±5.48  (-34.29-8.10)  1.12-3.99 | 6.79±4.23  (0.00-18.00)  5.69-7.90 | 11.90±7.04  (0.51-28.00)  10.06-13.75 | 14.44±8.02  (3.27-31.99)  12.34-16.54 |

| **(D)**  **Right femur** | **Max Dose (Gy)**  (n=53) | **V40 (%)**  (n=53) |
| --- | --- | --- |
| **Patients out of constraint** | 0 | 0 |
| **Mean±SD**  **(range)**  **IC95%** | 31.37±7.57  (0.00-46.67)  29.37-33.37 | 0.06±0.29  (0.00-2.10)  -0.02-0.13 |

| **(E)**  **Left femur** | **Max Dose (Gy)**  (n=54) | **V40 (%)**  (n=54) |
| --- | --- | --- |
| **Patients out of constraint** | 0 | 0 |
| **Mean±SD**  **(range)**  **IC95%** | 31.24±7.99  (0.00-51.16)  29.15-33.33 | 0.04±0.20  (0.00-1.40)  -0.01-0.09 |

| **(F)**  **Penis Bulb** | **Max Dose (Gy)**  (n=52) |
| --- | --- |
| **Patients out of constraint** | 21 |
| **Mean±SD**  **(range)**  **IC95%** | 44.10±21.77  (8.28-73.40)  38.19-50.02 |
